# Real-World Objects Are Represented in Visual Long-Term Memory Both as Unbound Features and as Bound Objects

**DOI:** 10.3389/fpsyg.2020.580667

**Published:** 2020-12-07

**Authors:** Christof Kuhbandner

**Affiliations:** Department of Psychology, University of Regensburg, Regensburg, Germany

**Keywords:** visual memory, feature memory, feature binding, object memory, perceptual long-term memory

Recent research has shown that observers store a vast amount of viewed real-world objects in visual long-term learning with high precision (e.g., Standing, 1973; Vogt and Magnussen, 2007; Brady et al., 2008), even when objects have been processed without any attention and intention of learning (Kuhbandner et al., 2017). However, one open issue that has attracted considerable attention recently is the nature of the stored visual long-term memory representations. Since objects are higher-level constructs that represent patterns of lower-level features, two contrasting views have been put forward: objects may be stored in in the form of sets of independent feature representations or in the form of unitary feature-bound object representations (e.g., Brady et al., 2013; van den Honert et al., 2017).

In two simultaneously published recent papers, contradictory conclusions are drawn. In a paper by Utochkin and Brady (2020), the authors conclude that objects are stored as sets of independent features, based on a series of experiments showing that object features are only weakly bound and can easily be unbound in long-term memory, a conclusion which is also supported by a previous study of Brady et al. suggesting that objects features are forgotten independently of each other (Brady et al., 2013). By contrast, in a paper by Balaban et al. (2019), the authors conclude that objects are stored as unitary feature-bound representations, based on a series of experiments following the protocol of the study by Brady et al. (2013; Experiment 2) but analyzing the data with an alternative analytical method, suggesting that object features are forgotten in a dependent manner.

At first glance, one could be tempted to conclude from such contradictory findings that further research is needed to clarify which view is actually correct. However, there is another possibility that is not considered in either of the two papers: it may be that visual information can be flexibly stored in visual long-term memory both feature-based and object-based, depending on the requirements of the current situation. If so, debates about whether visual objects are stored either feature-based or object-based may be misleading. Instead, the relevant question that should be explored in future research would be which factors determine whether objects are stored in visual long-term memory feature-based or object-based.

Such a theoretical assertion is based on two assumptions. First, it must be the case that real-world objects can be stored in visual long-term memory both feature-based and object-based. Second, it must be the case that the different storage formats have different functionalities and can be flexibly used. Regarding the first assumption, the contradictory findings reported in the papers by Brady et al. (2013), Balaban et al. (2019), and Utochkin and Brady (2020) can be taken as evidence that real-world objects can be stored both feature-based and object-based based. This is also supported by the fact that both in studies examining feature memory (e.g., Magnussen and Dyrnes, 1994; Magnussen et al., 2003) and in studies examining object memory (e.g., Ceraso et al., 1998; Walker and Cuthbert, 1998), the existence of long-lasting memory representations has been proven. The assumption that visual objects can be stored both feature-based and object-based is also found in prominent memory models such as the multiple-entry, modular memory framework (Johnson, 1983), postulating that there are feature-based and object-based memory subsystems. Furthermore, the existence of qualitatively different types of representational formats has also been recognized in theories about the hierarchical structure of visual memory (for a review, see, e.g., Brady et al., 2011). In fact, in research on visual working memory, it has been shown that both feature-based and object-based representations have to be assumed to fully explain the observed performance patterns (e.g., Fougnie et al., 2010, 2013).

To shed light on the different functionalities of feature-based and object-based memory representations, it is helpful to see how real-world objects are initially represented in the cognitive system during perception. Broadly speaking, two qualitatively different processing steps are involved (e.g., Tarr, 1995; Riesenhuber and Poggio, 1999; Serences and Yantis, 2006). First, visual features such as orientation, colors, and so forth, are extracted from the visual input, a process by which a representation of the visual input in terms of a collection of independent features is created. Second, informative features are recoded into bound object representations and uninformative features discounted, leading to the phenomenological experience of perceiving coherent objects. Importantly, object representations are not *ad hoc* formed independent of previous visual experiences. Rather, the recoding of features is informed by a stored inner model of the structure of the world that that reflects the current visual knowledge about objects derived from previous visual experiences.

As already postulated by Piaget (1970) and recently elaborated in theories about the so-called predictive brain (e.g., Clark, 2013), in order to allow adaptive learning, two opposing requirements have to be met by our visual system. On the one hand, to keep stability, incoming information has to be processed with respect to stored inner model of the world (assimilation). On the other hand, to allow adaptation, the current inner world model has to be continuously updated based on inconsistent incoming information (accommodation). The storing of feature-based vs. object-based memory representations may serve the fulfillment of these opposing requirements. As long as the current inner world model is appropriate, it can be imposed on incoming visual information so that visual experiences can be resource-efficiently stored as coherent objects based on current inner object models. Thus, object-based memory representations may serve the function of assimilation. In situations where the current inner world model does not sufficiently represent the incoming information, the inner model itself has to be updated based on the inconsistent information. In such a case, it would be functional to store visual experiences in the form of feature representations. Thus, feature-based memory representations may serve the function of accommodation (for an illustration, see Figure 1).

**Figure 1:**
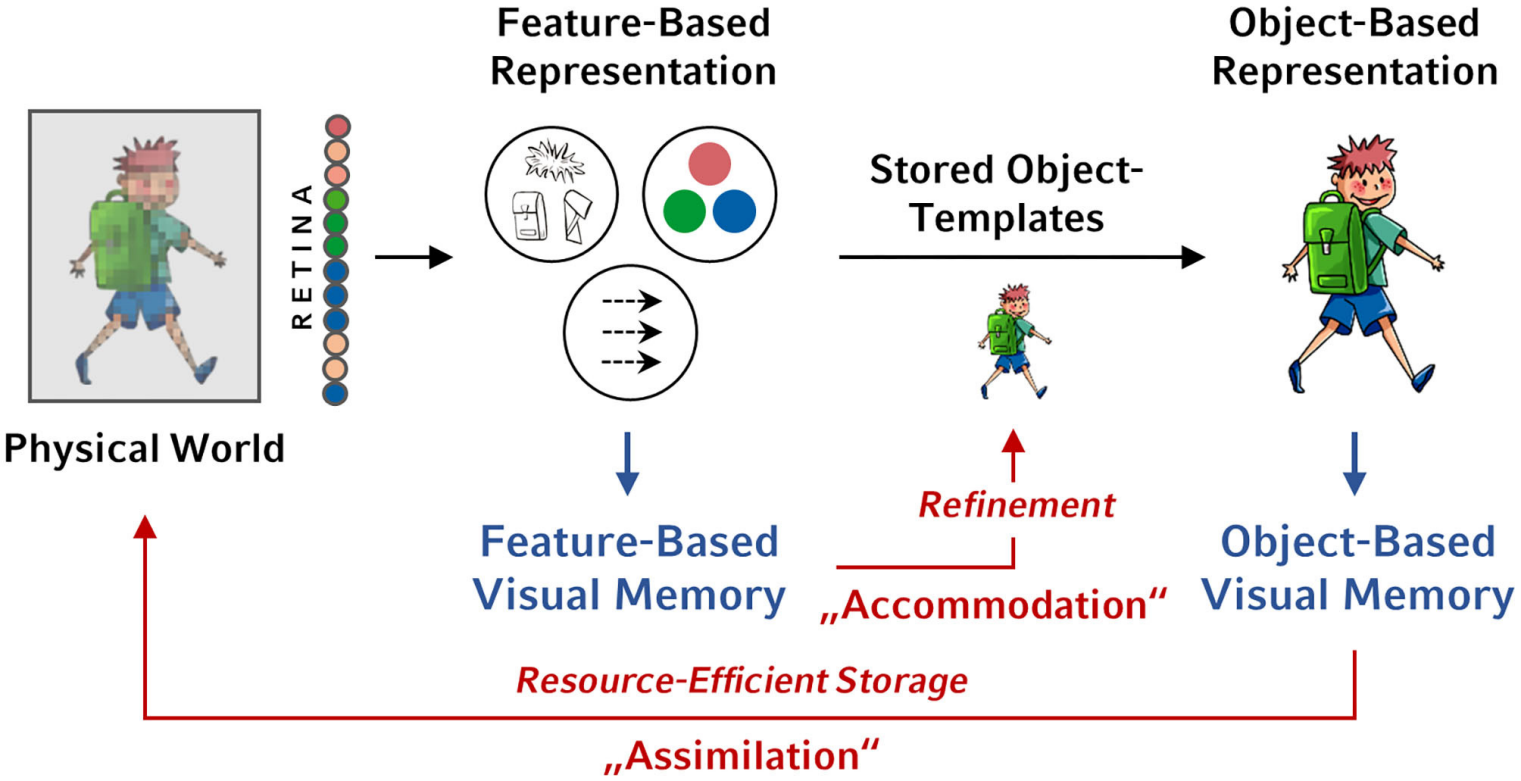
Illustration of the two types of visual long-term memory representations. During perception, at an initial step, the visual input is represented in terms of a collection of independent features such colors, orientations, and so forth. At a subsequent step, the features are recoded into bound object representations based on already stored object templates that reflect the currently stored inner model of the structure of the world derived from previous experiences. The outputs of both processing steps can be stored in memory, leading to unbound feature-based memory representations and to coherent object-based memory representations. From a functional perspective, as long as the current inner model of the structure of the world is appropriate, visual experiences can be resource-efficiently stored as coherent objects based on currently stored object-templates (“assimilation”). In situations where the current inner world model does not sufficiently represent the incoming information, feature-based memory representation can be used to refine the current inner model of the structure of the world (“accommodation”).

Therefore, depending on the appropriateness of the current inner model of the world, visual experiences may be stored either as unitary feature-bound objects representations (assimilation) or as independent feature representations (accommodation). A recent study examining the effect of affective state on the storing of real-world objects in visual long-term memory provides both behavioral and neurophysiological evidence that this is indeed the case (Spachtholz and Kuhbandner, 2017). As proposed in prominent theories on affect-cognition interactions (e.g., Clore and Huntsinger, 2007) and demonstrated in numerous studies (e.g., Fiedler et al., 2003; Kuhbandner et al., 2009), affect signals the validity of one's current inner model of the world, with positive affect validating and negative affect invalidating it, with the consequence that positive affect triggers processes of assimilation and negative affect processes of accommodation. Consistent with this, when real-world objects were encoded while experiencing positive affect, objects were more likely stored in the form of feature-bound object representations mediated by attention-related brain activities. By contrast, when real-world objects were encoded while experiencing negative affect, objects were more likely stored as independent feature representations mediated by pre-attentive brain activities.

Evidence for the assumption that the nature of memory representations is not unitary but a mixture of both feature-based and object-based representations is indeed also provided by the results of the studies by Balaban et al. (2019) and Utochkin and Brady (2020). For instance, in the study by Balaban et al. (2019), there is a number of memory reports where observers remember one feature but forget another one, indicating that memory representations are not purely object-based. Similarly, in the study by Utochkin and Brady (2020), interference is observed from irrelevant features when observers are asked to report relevant features, indicating that memory representations are not purely feature-based.

Taken together, the current state of research suggests that visual long-term memory is not a unitary system that stores real-world objects only in one specific representational format. Rather, real-world objects can be flexibly stored both as sets of independent features and as unitary feature-bound objects, depending on the requirements of the current situation. In particular, there may be a number of other factors beyond affect that influence the way real-world objects are stored in long-term memory. For instance, the storage format may vary as a function of the amount of previous experiences with encountered objects, making inner object models more or less appropriate. Furthermore, similar to affective state, current physical, motivational, and cognitive states may play important roles, which have been shown to systematically influence assimilation-accommodation tendencies as well (e.g., Leipold et al., 2014). At the interindividual level, individual habitual assimilation-accommodation tendencies may influence whether real-world objects are stored preferentially feature-based or object-based, and it may even be that there are cultural differences as suggested by studies showing that cultures systematically vary whether local or global visual information is favored during perception (e.g., Lao et al., 2013). Examining such factors may be more fruitful than trying to determine an illusionary unitary representational format of visual long-term memory.

## Author Contributions

The author confirms being the sole contributor of this work and has approved it for publication.

## Conflict of Interest

The author declares that the research was conducted in the absence of any commercial or financial relationships that could be construed as a potential conflict of interest.
